# Evaluation of the Pinhole Method Using Carbon Dioxide Laser on Facial Telangiectasia

**DOI:** 10.3390/jcm12082849

**Published:** 2023-04-13

**Authors:** Sang Seok Woo, Hongki Gwak, Seung Seog Han, In Suck Suh, Seong Hwan Kim

**Affiliations:** 1 Department of Plastic and Reconstructive Surgery, Kangnam Sacred Heart Hospital, Hallym University College of Medicine, Seoul 07441, Republic of Korea; 2 Division of Breast and Thyroid Surgical Oncology, Department of Surgery, Hwahong Hospital, Suwon 16630, Republic of Korea; 3 Department of Dermatology, I Dermatology Clinic, Seoul 08093, Republic of Korea

**Keywords:** facial telangiectasias, pinhole method, carbon dioxide laser, CO_2_ laser treatment

## Abstract

Facial telangiectasias are small, dilated blood vessels frequently located on the face. They are cosmetically disfiguring and require an effective solution. We aimed to investigate the effect of the pinhole method using a carbon dioxide (CO_2_) laser to treat facial telangiectasias. This study included 155 facial telangiectasia lesions in 72 patients who visited the Kangnam Sacred Heart Hospital, Hallym University. Treatment efficacy and improvement were evaluated by quantitative measurements performed by two trained evaluators who assessed the percentage of residual lesion length using the same tape measure. Lesions were evaluated before laser therapy and 1, 3, and 6 months after the first treatment. Based on the initial lesion length (100%), the average percentages of the residual length at 1, 3, and 6 months were 48.26% (*p* < 0.01), 4.25% (*p* < 0.01), and 1.41% (*p* < 0.01), respectively. Complications were evaluated using the Patient and Observer Scar Assessment Scale (POSAS). The average POSAS scores improved from 46.09 at the first visit to 23.42 (*p* < 0.01), and 15.24 (*p* < 0.01) at the 3- and 6-month follow-up. No recurrence was noted at the 6-month follow-up. CO_2_ laser treatment using the pinhole method to treat facial telangiectasias is a safe, inexpensive, and effective treatment that provides patients with excellent aesthetic satisfaction.

## 1. Introduction

Telangiectasia, a prominent capillary vasodilation often observed on the surface of the skin, varies in diameter between 0.1 and 1 mm. Its appearance can be clinically classified into simple or linear, spider form, or arboriform [1,2]. Telangiectasias occurring on the face can be a major cosmetic concern as they are more noticeable. Various treatment methods for facial telangiectasia, including cryotherapy, dye laser therapy, sclerotherapy, topical agents, oral estrogens, electrosurgery, and radioactive treatments, have been discussed in previous studies [1,2,3,4,5,6]. Their effectiveness has been studied. By comparing the action time, healing period, and possible side effects, appropriate treatment can be selected based on the condition of the lesion and suitability of the patient. Most of these treatments are effective with various advantages and disadvantages specific to each modality. For example, complications including telangiectatic matting, arterial ulceration, tissue necrosis, and hypertrophic scar formation have been reported [6,7,8,9,10,11,12]. In contrast, less invasive treatments showed recurrences [13].

The pinhole method involves creating multiple small holes that extend from the epidermis layer to the deeper dermis using an ultra-pulse carbon dioxide (CO_2_) laser. The effectiveness of this method in treating various skin conditions, such as hypertrophic scars, sebaceous hyperplasia, anetoderma, and elastosis perforans serpiginosa, has been proven [14,15,16,17,18,19]. In contrast with the conventional CO_2_ ablative laser, the pinhole method can induce regeneration and realignment of collagen bundles at the desired depth [14,15,16,17,18,19]. We hypothesized that this method could be an excellent option to treat facial telangiectasias of varying shapes and depth by reducing the appearance of dilated vessels with minimal adverse effects. This study aimed to investigate the effect of the pinhole method using a CO_2_ laser to treat facial telangiectasias. This was achieved by measuring the lengths of residual lesions and evaluating the overall outcome and further complications with scar assessment.

## 2. Materials and Methods

This retrospective study was approved by the Institutional Review Board (Kangnam Sacred Heart Hospital Institutional Review Board, IRB number 2022-06-012). All procedures involving human participants were performed in accordance with the ethical standards established by the institutional and/or national research committee and with the 1964 Helsinki Declaration and its later amendments or comparable ethical standards.

### 2.1. Patient Selection and Laser Methods

The clinical records of 72 patients (with a total of 155 facial telangiectasia lesions) who visited our clinic between April 2018 and August 2021 were reviewed. Patients with facial telangiectasias were treated with a CO_2_ laser using the pinhole method for 2–3 sessions at 1-month intervals. The patients were of Northeast Asian origin, 35 men and 37 women, aged 43–78 years, and had Fitzpatrick skin types III–IV.

The exclusion criteria included additional treatment as well as laser treatment, a history of hypertrophic scarring or keloids, photosensitivity, pigmentation due to recent exposure to sunlight, a follow-up loss of fewer than three times for treatment, pregnancy, and immunosuppressive drug use.

We used the pinhole method with an ultra-pulse CO_2_ laser (UM-L30^®^, Union Medical Co. Ltd., Seoul, Republic of Korea) to treat the lesions. All laser treatments were performed by the same plastic surgeon.

Thirty minutes before the treatment, 5% lidocaine/prilocaine cream (EMLA 5% cream, AstraZeneca, London, United Kingdom) was applied to the treatment area with film dressing coverage. After treatment, topical antibiotic ointment was applied to minimize edema and infection. The patients were instructed to avoid exposure to sunlight for 3 months.

**Figure 1:**
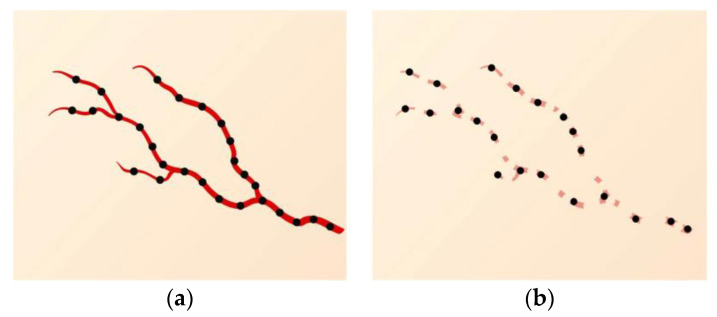
(**a**) Multiple black dots are the treated areas at telangiectasia. (**b**) The residual areas of the lesion are treated in the second session in the same manner.

Laser fluencies were delivered under the following settings: spot diameter, 1 mm; pulse duration, 200 ms; and peak power, 20 W. In the first session, multiple small holes were made along the entire lesion at 1–2 mm intervals to the depth of the papillary dermis, where the telangiectatic vessels existed as illustrated in Figure 1. The residual areas of the lesion were treated in the second and third sessions in the same manner.

### 2.2. Outcome Assessment

The clinical response to the treatment and its complications were evaluated. Treatment efficacy and improvement were evaluated with quantitative measurements by comparing the length of lesion before treatment and 1, 3, and 6 months after the first session. Digital photographs of the lesions were obtained with a length measurement under the same light source and illumination conditions using a standard light source box. Since the length of the lesion was different for each case, the ratio of the original length to the length of the remaining lesion was measured. Quantitative evaluation was performed by assessing the percentage of residual lesion length measured with the same tape measure.

The overall outcome and complication was assessed using the Patient and Observer Scar Assessment Scale (POSAS) before treatment and 3 and 6 months after the first session. Two evaluators, who were trained to score in a similar manner, assessed the patients during every visit.

The POSAS is composed of two numerical scales that evaluate the signs and symptoms of healing with two parts: scales for patients and for observers [20,21]. Both contain six items punctuated numerically from 1 to 10, which comprise the “total score” of the scale for both the patient and observer. The lowest score is 1, which corresponds to a normal skin condition. The total score of both scales can be calculated simply by adding the scores for each of the six items [20,21]. Each item evaluates a specific parameter. The Patient Scar Assessment Scale includes pain, itching, color, stiffness, thickness, and relief. The Observer Scar Assessment Scale includes vascularity, pigmentation, thickness, relief, pliability, and surface area. The total score ranges from 6 to 60 [20,21].

Category boxes are provided to assess categories qualitatively and quantitatively. In this way, the gravity and direction of disorder are addressed. The items in these categories are not included in the total POSAS score, even though they are considered clinically relevant for complete documentation [20,21].

In addition, the patient and observer record their “general opinion” on the appearance of the lesion regardless of the “total score.” The 10-point scale is further used for the “general opinion,” with 10 being the worst imaginable.

### 2.3. Statistical Analysis

Repeated measures analysis of variance (ANOVA) was used to determine if the therapy resulted in improvements. The reduced length of the lesion was evaluated, and scar scale scores from the first visit to 6 months after treatment were compared. The results were analyzed using IBM SPSS Statistics for Windows (version 22.0; IBM, Armonk, NY, USA). Statistical significance was defined as *p* < 0.01.

## 3. Results

### 3.1. Patients

In total, 72 patients were included in the study. Treatments were performed on 104 lesions in 35 men and 51 lesions in 37 women, respectively. The mean age of patients was 58.3 ± 12.9 and 43.7 ± 13.1 years for men and women, respectively. The most common anatomical areas with visible telangiectasias were the cheeks and nasal area. The average lengths of the lesions were 12.3 ± 3.8 mm in males and 10.8 ± 2.0 mm in females. The basic patient demographics, including age, sex, and location, are listed in Table 1.

### 3.2. Quantitative Evaluation (Objective)

**Figure 2:**
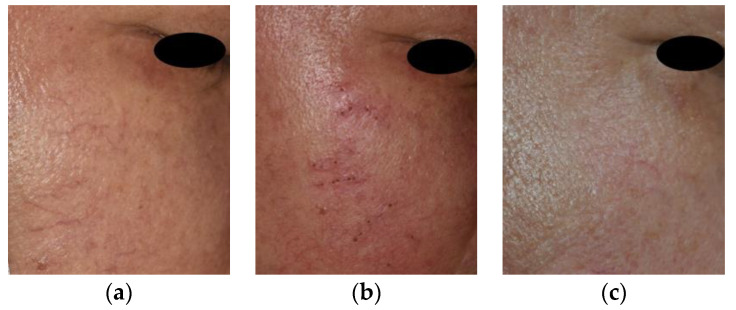
Facial telangiectasia in a 57-year-old man. (**a**) Telangiectasia on the right cheek before treatment. (**b**) Multiple deep holes were made in line with the course of the telangiectasia. (**c**) The telangiectasia showed significant improvement 3-month follow-up.

**Figure 3:**
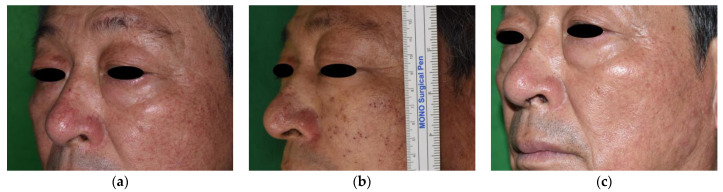
Facial telangiectasia in a 68-year-old man. (**a**) Telangiectasia on nasal dorsum, left cheek before treatment. (**b**) Multiple deep holes were made in line with the course of the telangiectasia. (**c**) The telangiectasia showed improvement on left cheek, but traces of lesion left on nasal dorsum were observed at 3-month follow-up.

Two patients with three lesions were lost to follow-up at the 3-month evaluation, and an additional 5 patients with 12 lesions were lost to follow-up during the 6-month evaluation, leaving a total of 65 patients with 140 lesions at the 6-month evaluation. The average length of the lesions before treatment was 11.8 ± 3.4 mm. The average size of the lesions significantly decreased to 5.7 ± 1.9 mm after 1 month, 0.9 ± 1.0 mm after 3 months, and 0.2 ± 0.7 mm after 6 months (*p* < 0.01). The change in average lesion size after the procedure is described in Table 2. Most lesions disappeared 2 months after the first treatment session, which is shown in Figure 2. Trace lesions (mostly 1–2 mm in length) were observed in two male and three female patients at 3-month follow-up as shown in Figure 3. These eventually disappeared at 6 months.

### 3.3. The Patient and Observer Scar Assessment Scale (Subjective)

Vascularity, pigmentation, and relief were the subcategories with the top three highest scores on the observer scale. They were initially scored at 6.6 ± 1.7, 6.6 ± 1.1, and 6.5 ± 1.2, respectively. The scores decreased at 3- and 6-month follow-up by 1.92 ± 0.84 (*p* < 0.01), 1.96 ± 0.85 (*p* < 0.01), and 1.52 ± 0.54 (*p* < 0.01) and 1.31 ± 0.46 (*p* < 0.01), 1.36 ± 0.48 (*p* < 0.01), and 1.00 ± 0.00 (*p* < 0.01), respectively. Color difference and irregularity were the categories with the top two highest scores on the patient scale. They were scored at 8.4 ± 1.2 and 7.1 ± 0.8, respectively. The scores decreased at 3- and 6-month follow-up by 5.0 ± 0.8 (*p* < 0.01) and 4.0 ± 0.9 (*p* < 0.01) and 1.5 ± 0.5 (*p* < 0.01) and 1.5 ± 0.5 (*p* < 0.01), respectively. The results demonstrated improvement over time with an increase in the number of treatments. The *p*-value of most subcategories was <0.01. The POSAS score is described in Table 3.

### 3.4. Complications

**Figure 4:**
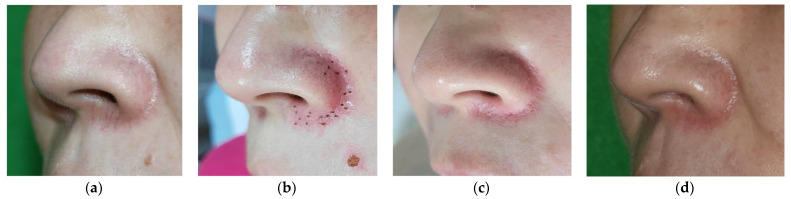
Facial telangiectasia in a 45-year-old man. (**a**) Telangiectasia on the nasal alar area before treatment. (**b**) Multiple deep holes were made in line with the course of the telangiectasia. (**c**) The telangiectasia showed significant improvement 1 month after the first treatment session with the pinhole method using the CO_2_ laser. However, a slight amount of hyperpigmentation was noted. (**d**) Hyperpigmentation was slightly improved at 6-month follow-up.

Overall, no significant adverse effects, including infection or bleeding, were observed during the follow-up period. In five male patients, a slight amount of hyperpigmentation was noted compared to female patients in the first 3-month follow-up period, which gradually improved at 6-month follow-up as shown in Figure 4. However, the patient satisfaction scores of male patients were remarkably higher than those of female patients. This may be because the proportion of elderly patients was relatively higher in the male patient group.

## 4. Discussion

To the best of our knowledge, this is the first study to retrospectively analyze and compare the result of the CO_2_ laser using the pinhole method for facial telangiectasis to demonstrate its effectiveness and safety. This study showed a cure rate of 98.6%, which shows its non-inferiority compared to the treatment effect of 50–100% of existing treatments [3,7,22,23,24].

Telangiectasia is a capillary vasodilation that is easily visible on various skin areas. It can be broadly classified into tortuous veins, with diameters varying from less than 1 mm to more than 5 mm, with those less than 1 mm referred to as telangiectasia [25,26,27,28].

Recent advances in laser therapy have demonstrated particularly good outcomes and high satisfaction in patients with facial telangiectasias [3,7,29]. The basic concept of laser technology, selective photothermolysis, and chromophores is to localize the thermal damage to the lesion and minimize collateral thermal damage to the surrounding tissue by selecting the appropriate wavelength of light that the specific targeted chromophore will absorb [3,7,29]. In telangiectasias, the intended chromophore is mostly intravascular oxyhemoglobin [3,7,29]. The thermal energy is transferred to the surrounding blood vessel walls, resulting in destruction of the targeted vessels [3,7,29].

The potassium titanyl phosphate laser (KTP, 532 nm), pulsed dye lasers (PDL, 585 nm and 595 nm), and intense pulsed light systems (IPL, 500–1200 nm) have become the standard treatment for facial telangiectasia because of their superior clinical efficacy and patient safety record [3,7,24,29,30]. PDL was the first laser device developed that utilized the concept of selective photothermolysis to manage vascular lesions [3,23]. Despite its proven therapeutic efficacy and safety profile, patient acceptance of this treatment option may be limited by the appearance of purpura at the treatment site, which can persist for 7–14 days [7]. Moreover, techniques to prevent purpura have been developed but are often less effective than the high-fluence PDL [24]. In 2016, Guida et al. reported reappearance after clearance with successful laser treatment using a flashlamp-pumped PDL [13].

Furthermore, other laser treatments have been used to achieve successful therapeutic effects [3,7,29]. However, drawbacks and the possibility of recurrence remain. The primary target chromophore, oxyhemoglobin, is a normal, oxygen-carrying form of hemoglobin not exclusive to the telangiectatic capillary [3,7,29]. Hence, selective thermal damage only in the desired capillaries is impossible without damaging the surrounding normal tissues. In contrast, the pinhole method uses an ablative 10,600 nm CO_2_ laser that is absorbed by water and transfers heat energy by evaporation [3,7,29]. As most soft tissues contain water molecules, this laser cannot provide selective photolysis [14,15,16,17,18,19]. However, the physician can selectively irradiate multiple small holes manually. Due to these characteristics, we expect this method to be effective in treating facial telangiectasias.

We applied the previously unreported pinhole-type CO_2_ laser method to patients with telangiectasia and achieved good results without any unusual complications. The pinhole method, also called manual-type fractionated laser treatment, is a novel method that involves the use of a conventional ablative laser [14,15,16,17,18,19]. The pinhole method with ablative lasers involves creating multiple small holes that penetrate the skin from the epidermis layer to the deeper dermis layer at 1 to 3 mm intervals [14,15,16,17,18,19]. In cases of telangiectasia, treating only some points with the pinhole technique is sufficient for treating the entire affected surface. Blood vessels are analogous to pipes, and the coagulated, obstructed points can stop the functioning of the entire structure [14].

In addition, there is a tendency for bleeding due to the characteristics of telangiectasia lesions [14]. Bleeding control is not possible when treating these lesions with other laser devices, and manual compression is required in some cases [4]. However, in the case of the CO_2_ lasers, hemostasis can be achieved, and compression may not be required [14].

CO_2_ lasers are some of the most popular laser devices used to treat skin lesions. The pinhole method can be easily applied for this laser as it does not require additional equipment. Accordingly, this method has been used to treat various skin diseases, such as burn scars, poorly oriented scars, secondary anetoderma, elastosis perforans, serpiginosa, and syringoma [14,15,16,17,18,19]. With CO_2_ laser treatment via the pinhole method, laser energy can be delivered to the deep dermis layer to destroy target dermal tissues and achieve thermal stimulation of surrounding collagen bundles and elastic fibers [18]. This results in the textural improvement of skin lesions [18].

The pinhole method is easy to perform and can be implemented simply using a CO_2_ laser. CO_2_ lasers require the most basic equipment of all skin lasers, and the equipment itself is inexpensive compared to other laser devices.

Our results mostly demonstrated excellent improvement, but we could not confirm differences in a few subcategories. These results were due to the small sample size and limitations of subcategories assessed by the scar scale, which are not appropriate for skin lesions. For example, “surface area” indicates the degree of wideness or contraction of a scar, which is usually absent in skin lesions. In addition, “pliability” or “stiffness” is a subjective measure and did not differ significantly; therefore, the scores of those subcategories were observed to rather increase in some cases.

A limitation of our study is that, as it was a retrospective study conducted in a single clinical setting, there was no control group, which may mean it there is insufficient evidence to generalize the applicability of this treatment. However, our results are strengthened by repeated ANOVA and the respective comparison with the results of previous studies. Further studies are required for accurate comparison.

## 5. Conclusions

The treatment of facial telangiectasia using the pinhole technique with ablative lasers is safe, cheap, and easily accessible and is an effective procedure. Therefore, the CO_2_ laser pinhole method is recommended as the primary treatment option for facial telangiectasia.

## Figures and Tables

**Table 1:** Patients’ demographics and characteristics of skin lesions.

	Male (*n* = 35)	Female (*n* = 37)	Total (*n* = 72)
Age (years)	58.1 (23–78)	44.2 (24–75)	53.5 (23–78)
Lesions	104	51	155
Length (mm)	12.3 ± 3.8	10.8 ± 2.0	11.8 ± 3.4
Location *n* (%)			
Forehead	9 (8.7)	6 (11.8)	15 (9.7)
Glabella	0	1 (2.0)	1 (0.6)
Eyebrow	1 (1.0)	0	1 (0.6)
Eyelid	2 (1.9)	3 (5.9)	5
Temporal area	3 (2.9)	0	3
Cheek	32 (30.8)	11(21.6)	43
Nose	46 (44.2)	22 (43.1)	68
Philtrum	7 (6.7)	1 (2.0)	8
Chin	2 (1.9)	4 (7.8)	6
Submandibular area	2 (1.9)	3 (5.9)	5

**Table 2:** Change in the size of the lesion after CO_2_ laser treatment.

	Length of Lesion (mm)				
	0 Month	1 Month	3 Months	6 Months	*p*-Value *
Total (*n* = 155)	11.8 ± 3.4	5.7 ± 1.9	0.9 ± 1.0	0.2 ± 0.7	<0.01
Male (*n* = 104)	12.3 ± 3.8	6.1 ± 0.9	0.6 ± 0.9	0.2 ± 0.5	<0.01
Female (*n* = 51)	10.8 ± 2.0	5.0 ± 0.2	0.3 ± 0.7	0.1 ± 0.2	<0.01
FU patient (lesions)	72 (155)	72 (155)	70 (152)	65 (140)	

* *p*-Value: number of treatments and the result (repeated measures ANOVA).

**Table 3:** POSAS score change after treatment.

		Scale Score			
		0 Month	3 Months	6 Months	*p*-Value *
OSAS	Vascularity	6.6 ± 1.7	1.9 ± 0.8	1.3 ± 0.5	<0.01
	Pigmentation	6.6 ± 1.1	2.0 ± 0.9	1.4 ± 0.5	<0.01
	Thickness	1.5 ± 0.5	1.0 ± 0	1.0 ± 0	<0.01
	Relief (irregularity)	6.5 ± 1.2	1.5 ± 0.5	1.0 ± 0	<0.01
	Pliability	2.1 ± 0.8	2.0 ± 0.8	2.0 ± 0.8	0.381
	Surface area	1.0 ± 0	1.0 ± 0	1.0 ± 0	-
	Total score	24.3 ± 2.5	9.4 ± 1.5	7.6 ± 0.9	<0.01
PSAS	Pain	1.5 ± 0.5	1.0 ± 0	1.0 ± 0	<0.01
	Itchiness	2.0 ± 0.9	1.0 ± 0	1.0 ± 0	<0.01
	Color difference	8.4 ± 1.2	5.0 ± 0.8	1.5 ± 0.5	<0.01
	Stiffness	1.4 ± 0.5	1.5 ± 0.5	1.4 ± 0.5	-
	Thickness	1.5 ± 0.5	1.5 ± 0.5	1.2 ± 0.4	<0.01
	Irregularity	7.1 ± 0.8	4.0 ± 0.9	1.5 ± 0.5	<0.01
	Total score	21.8 ± 1.9	14.0 ± 1.4	7.6 ± 0.9	<0.01
POSAS	Total score	46.1 ± 3.3	23.4 ± 2.8	15.2 ± 1.8	<0.01

OSAS, Observer Scar Assessment Scale; PSAS, Patient Scar Assessment Scale. * *p*-value: number of treatments and the result (repeated measures ANOVA).

## Data Availability

The data presented in this study are available on request from the corresponding author.

## References

[1] Gao L., Qu H., Gao N., Li K., Dang E., Tan W., Wang G. (2020). A retrospective analysis for facial telangiectasia treatment using pulsed dye laser and intense pulsed light configured with different wavelength bands. J. Cosmet. Dermatol..

[2] Mekic S., Hamer M.A., Wigmann C., Gunn D.A., Kayser M., Jacobs L.C., Schikowski T., Nijsten T., Pardo L.M. (2020). Epidemiology and determinants of facial telangiectasia: A cross-sectional study. J. Eur. Acad. Dermatol. Venereol..

[3] Hare McCoppin H.H., Goldberg D.J. (2010). Laser treatment of facial telangiectases: An update. Dermatol. Surg..

[4] Lee S.J., No Y.A., Kang J.M., Chung W.S., Kim Y.K., Seo S.J., Park K.Y. (2016). Treatment of hesitation marks on the forearm by the pinhole method. Lasers Med. Sci..

[5] Andrews R.H., Dixon R.G. (2021). Ambulatory Phlebectomy and Sclerotherapy as Tools for the Treatment of Varicose Veins and Telangiectasias. Semin. Interv. Radiol..

[6] Wittens C., Davies A.H., Bækgaard N., Broholm R., Cavezzi A., Chastanet S., de Wolf M., Eggen C., Giannoukas A., Gohel M. (2015). Editor’s Choice—Management of Chronic Venous Disease: Clinical Practice Guidelines of the European Society for Vascular Surgery (ESVS). Eur. J. Vasc. Endovasc. Surg..

[7] Kauvar A.N., Khrom T. (2005). Laser treatment of leg veins. Semin. Cutan. Med. Surg..

[8] Olivencia J.A. (1997). Complications of ambulatory phlebectomy. Review of 1000 consecutive cases. Dermatol. Surg..

[9] Ramelet A.A. (1997). Complications of ambulatory phlebectomy. Dermatol. Surg..

[10] Bergan J.J., Weiss R.A., Goldman M.P. (2000). Extensive tissue necrosis following high-concentration sclerotherapy for varicose veins. Dermatol. Surg..

[11] Goldman M.P., Sadick N.S., Weiss R.A. (1995). Cutaneous necrosis, telangiectatic matting, and hyperpigmentation following sclerotherapy. Etiology, prevention, and treatment. Dermatol. Surg..

[12] Davis L.T., Duffy D.M. (1990). Determination of incidence and risk factors for postsclerotherapy telangiectatic matting of the lower extremity: A retrospective analysis. J. Dermatol. Surg. Oncol..

[13] Guida S., Galimberti M.G., Bencini M., Pellacani G., Bencini P.L. (2017). Telangiectasia of the face: Risk factors for reappearance in patients treated with dye laser. J. Eur. Acad. Dermatol. Venereol..

[14] Railan D., Parlette E.C., Uebelhoer N.S., Rohrer T.E. (2006). Laser treatment of vascular lesions. Clin. Dermatol..

[15] Chung B.Y., Han S.S., Moon H.R., Lee M.W., Chang S.E. (2014). Treatment with the pinhole technique using erbium-doped yttrium aluminium garnet laser for a cafe au lait macule and carbon dioxide laser for facial telangiectasia. Ann. Dermatol..

[16] Kim J.H., Park H.Y., Lee W.S., Kang J.S. (2013). Sebaceous hyperplasia effectively improved by the pin-hole technique with squeezing. Ann. Dermatol..

[17] Yang J.H., Han S.S., Won C.H., Chang S.E., Lee M.W., Choi J.H., Moon K.C. (2011). Treatment of elastosis perforans serpiginosa with the pinhole method using a carbon dioxide laser. Dermatol. Surg..

[18] Lee S.J., Cho S., Kim Y.K., Cho S.B. (2013). Scar revision by the pinhole method using a 10,600-nm carbon dioxide laser. J. Cosmet. Laser Ther..

[19] Lee S.M., Kim Y.J., Chang S.E. (2012). Pinhole carbon dioxide laser treatment of secondary anetoderma associated with juvenile xanthogranuloma. Dermatol. Surg..

[20] Fearmonti R., Bond J., Erdmann D., Levinson H. (2010). A review of scar scales and scar measuring devices. Eplasty.

[21] Draaijers L.J., Tempelman F.R., Botman Y.A., Tuinebreijer W.E., Middelkoop E., Kreis R.W., Van Zuijlen P.P. (2004). The patient and observer scar assessment scale: A reliable and feasible tool for scar evaluation. Plast. Reconstr. Surg..

[22] Cassuto D.A., Ancona D.M., Emanuelli G. (2000). Treatment of facial telangiectasias with a diode-pumped Nd:YAG laser at 532 nm. J. Cutan. Laser Ther..

[23] Garden J.M., Bakus A.D. (1993). Clinical efficacy of the pulsed dye laser in the treatment of vascular lesions. J. Dermatol. Surg. Oncol..

[24] Alam M., Dover J.S., Arndt K.A. (2003). Treatment of facial telangiectasia with variable-pulse high-fluence pulsed-dye laser: Comparison of efficacy with fluences immediately above and below the purpura threshold. Dermatol. Surg..

[25] Kabnick L.S., Ombrellino M. (2005). Ambulatory phlebectomy. Semin. Interv. Radiol..

[26] Bowes L.E., Goldman M.P. (2002). Sclerotherapy of reticular and telangiectatic veins of the face, hands, and chest. Dermatol. Surg..

[27] Goldman M.P., Bergan J.J. (2007). Sclerotherapy: Treatment of Varicose and Telangiectatic Leg Veins.

[28] Goldman P.M. (1989). Polidocanol (aethoxysklerol) for sclerotherapy of superficial venules and telangiectasias. J. Dermatol. Surg. Oncol..

[29] Anderson R.R., Parrish J.A. (1983). Selective photothermolysis: Precise microsurgery by selective absorption of pulsed radiation. Science.

[30] Piccolo D., Crisman G., Kostaki D., Cannarozzo G., Sannino M., Chimenti S. (2016). Rhodamine intense pulsed light versus conventional intense pulsed light for facial telangiectasias. J. Cosmet. Laser Ther..

